# Knowledge‐based planning in robotic intracranial stereotactic radiosurgery treatments

**DOI:** 10.1002/acm2.13173

**Published:** 2021-02-09

**Authors:** Suhong Yu, Huijun Xu, Yin Zhang, Xin Zhang, Michael A. Dyer, Ariel E. Hirsch, Minh Tam Truong, Heming Zhen

**Affiliations:** ^1^ Department of Radiation Oncology Boston Medical Center Boston University school of Medicine Boston MA USA; ^2^ Department of Radiation Oncology University of Massachusetts Medical School Worcester MA USA; ^3^ Department of Radiation Oncology University of Maryland School of Medicine Baltimore MD USA; ^4^ Department of Radiation Oncology Rutgers‐Cancer Institute of New Jersey Rutgers‐Robert Wood Johnson Medical School New Brunswick NJ USA

**Keywords:** Cyberknife, knowledge‐based planning, stereotactic radiosurgery, stereotactic radiotherapy

## Abstract

**Purpose:**

To develop a knowledge‐based planning (KBP) model that predicts dosimetric indices and facilitates planning in CyberKnife intracranial stereotactic radiosurgery/radiotherapy (SRS/SRT).

**Methods:**

Forty CyberKnife SRS/SRT plans were retrospectively used to build a linear KBP model which correlated the equivalent radius of the PTV (r_eq_PTV_) and the equivalent radius of volume that receives a set of prescription dose (r_eq_Vi_, where V_i_ = V_10%_, V_20%_ … V_120%_). To evaluate the model’s predictability, a fourfold cross‐validation was performed for dosimetric indices such as gradient measure (GM) and brain V_50%_. The accuracy of the prediction was quantified by the mean and the standard deviation of the difference between planned and predicted values, (i.e., ΔGM = GM_pred_ − GM_clin_ and fractional ΔV_50%_ = (V_50%pred_ − V_50%clin_)/V_50%clin_) and a coefficient of determination, R^2^. Then, the KBP model was incorporated into the planning for another 22 clinical cases. The training plans and the KBP test plans were compared in terms of the new conformity index (nCI) as well as the planning efficiency.

**Results:**

Our KBP model showed desirable predictability. For the 40 training plans, the average prediction error from cross‐validation was only 0.36 ± 0.06 mm for ΔGM, and 0.12 ± 0.08 for ΔV_50%_. The R^2^ for the linear fit between r_eq_PTV_ and r_eq_vi_ was 0.985 ± 0.019 for isodose volumes ranging from V_10%_ to V_120%_; particularly, R^2^ = 0.995 for V_50%_ and R^2^ = 0.997 for V_100%_. Compared to the training plans, our KBP test plan nCI was improved from 1.31 ± 0.15 to 1.15 ± 0.08 (*P* < 0.0001). The efficient automatic generation of the optimization constraints by using our model requested no or little planner’s intervention.

**Conclusion:**

We demonstrated a linear KBP based on PTV volumes that accurately predicts CyberKnife SRS/SRT planning dosimetric indices and greatly helps achieve superior plan quality and planning efficiency.

## 1 Introduction

Stereotactic radiosurgery (SRS) and stereotactic radiotherapy (SRT) are advanced and highly precise forms of radiation therapy. They have been clinically used to treat intracranial tumors and functional abnormalities of the brain.^1^, ^2^, ^3^, ^4^ In contrast to conventional fractionated radiation therapy; SRS/SRT delivers one or a few fractions of large ablative dose to a relatively smaller target volume with sub‐millimeter target localization accuracy.^5^, ^6^, ^7^ The normal tissue sparing for the surrounding brain tissues is achieved by a very steep dose falloff outside the target regions. Favorable treatment results for brain tumors, for example, meningioma have been obtained using SRS/SRT.^4^, ^8^, ^9^, ^10^, ^11^, ^12^, ^13^, ^14^, ^15^ The dedicated machines designed for effective SRS and SRT include Gammaknife, conventional linear accelerator, and CyberKnife, which is a compact, image‐guided linear accelerator with a robotic manipulator.

Treatment planning system (TPS) of Cyberknife SRS/SRT such as Multiplan (Accuray Inc., Sunnyvale, CA) cooperates optimizes inverse treatment planning with the full function of Cyberknife for the accurate and versatile SRS/ SRT system. Depending on the complexity of the patient case, however, planning can be very time‐consuming.^16^ Also, the knowledge and experience of the planner in this complex technology is essential for the quality of treatment planning.^17^ The variation in Cyberknife manual planning between institutions and planners is a potential issue in terms of a consistent and high treatment quality of SRS/SRT.

Knowledge‐based planning (KBP) is a promising technique to tackle the challenges of planning efficiency and variation. KBP learns from the database of past clinical plans and captures clinician knowledge and experience in terms of rules and algorithms. KBP offers a shift toward the direction of treatment planning automation, standardized plan quality, and improved treatment planning efficiency. According to a recent review article,^18^ more than 70 papers in data‐driven KBP have been published for various IMRT technologies such as VMAT and Tomotherapy. Methods of knowledge models have been developed for predicting such parameters as dosimetric and dose–volume points, voxel‐level doses, and objective function weights. To allow the investigation of KBP in numerous clinical applications, some model development has led to commercial products in multiple TPSs including RapidPlan (Eclipse, Varian Medical Systems, Palo Alto, CA, USA),^19^, ^20^ Erasmus‐iCycle (Monaco, Elekta, Crawly, UK),^21^ and PlanIQ (Sun Nuclear, Melbourne, Florida, USA).^22^ For various cancer sites^23^, ^24^, ^25^, ^26^, ^27^ like brain, head and neck, spine, lung, prostate, etc,^19^, ^28^, ^29^ the benefits of KBP have been demonstrated in achieving comparable or improved planning quality, reduced planning time, and plan quality variation.

Particular for intracranial SRS/SRT, several groups have successfully developed KBP models for plan evaluation and optimization.^16^, ^30^, ^31^ Shiraishi *et al* ^30^ developed a comprehensive KBP method for achievable DVH prediction. Their quality metric estimation helps identify suboptimal treatment plans and guides the target objectives for the plan optimization. Ziemer et al^26^ proposed a KBP for planning automation using artificial neural network. They found that KBP yielded an equivalent or better planned compared to the manual planning. However, none of these studies applies to Cyberknife. Therefore, KBP for Cyberknife intracranial SRS/SRT planning is still lacking.

This study evaluates a KBP model for CyberKnife SRS/SRT treatments. We demonstrate a rigorous method to derive the "empirical values" that meets the clinic‐specific needs for different clinical settings and plan constraints. Our model predicts dosimetric indices which facilitate the automatic generation of shell constraints for isodose tuning and yield a highly efficient automated planning process. Here, we aim to extend KBP’s clinical benefit to CyberKnife intracranial patients.

## 2 Materials and Methods

This study was an Institutional Review Board (IRB)‐exempted retrospective review of intracranial SRS/SRT plans from 2017 to 2019 at Boston Medical Center. From April 2017 to January 2018, 40 consecutive intracranial SRS/SRT treated plans (26 patients) were selected for the initial analysis. Prescription dose (Rx) range was 15 Gy to 30 Gy in 1 or 5 fractions. Disease types included 35 brain metastases, 4 meningioma, and 1 glioblastoma (GBM). Seven of the total 40 plans (18%) had planning target volume (PTV) near (<1 cm) or overlapping with brainstem. One of the seven patients had right cochlear within 1cm. In the other 33 plans, PTV was more than 5cm away from critical organs at risk (OARs) (Table 1).

**TABLE 1:** Model plan characteristics.

Prescription	Number of plans *n* (%) (N = 40)	PTV volumes Mean (cc)	Number of Plans with OAR involvement
20Gy x 1	17 (42.5%)	0.82 (range: 0.05‐4.28)	1
18Gy x 1	7 (17.5)	1.84 (range: 0.02‐4.11)	1
15Gy x 1	1 (2.5%)	0.03	0
6Gy x 5	8 (20%)	21.91 (range: 1.44‐74.17)	1
5Gy x 5	7 (17.5%)	9.63 (range: 0.36‐23.18)	4

All the plans were created with Accuray TPS Multiplan version 4.6.1 using sequential optimization. Per our clinical practice, one to two fixed circular collimators were used according to the TPS conformality automatic selection based on the size of the PTV. The diameter of the bigger collimator around 2/3 or equal to the maximum PTV dimensions. The diameter of the smaller collimator is about the size of the minimum dimension of the PTV. Three to four shell constraints were applied, and additional OAR constraints were added when necessary. The size of the shells varied with different planners: In general, two smaller shells (like 1–2 mm and 6–8 mm) were used for high dose falloff, and one to two bigger shells for the low dose spreading (50% dose or lower). The sizes of larger shells (i.e., 10–20 mm or 30–50 mm) depend on the sizes of PTVs. The maximum constraints for PTVs were chosen as 200% of the RX. For the patients with multiple targets, each target yielded a separate plan. The target coverage goal was to cover at least 95% of the PTV volume with the prescription dose. All the final dose distributions were calculated using Raytracing algorithm with high resolution.

### 2.A Univariate regressions

A linear model using univariate regression was built for the 40 training plans. The coefficients of determinants, R^2^, were used as a measure of goodness of fit. The model correlated the equivalent radius of the PTVs (r_eq_PTV_) and the equivalent radius of volume receiving a set of percentage of the prescription dose (r_eq_Vi_, where V_i_ = V_10%_, V_20%_ … V_120%_).

(1) $$r_{eq\_Vi}=\alpha_{i}r_{eq\_PTV}+\beta_{i}$$

where α and β referred to the slope and offset of the fitted line, and r_eq_vi_ and r_eq_PTV_ are the radius of a sphere with geometric volume equals of V_i_ and PTV, respectively. Once α and β are obtained from the model, various dose volumes can be predicted for a given PTV volume.

### 2.B Model Validation and Prediction evaluation

The plan quality metrics such as conformity index (CI) and gradient measure (GM) are commonly used to evaluate intracranial stereotactic radiotherapy plans. GM is defined as.

(2) $$GM={\left(\frac{3}{4\pi }\right)}^{\frac{1}{3}}\left[V_{50\%}^{\frac{1}{3}}‐V_{100\%}^{\frac{1}{3}}\right]$$

The new conformity index (nCI) used in this study is defined as.

(3) $$nCI=\frac{\mathrm{TV}}{{\mathrm{TV}}_{\mathrm{RX}}}X\frac{V_{\mathrm{RX}}}{{\mathrm{TV}}_{\mathrm{RX}}}$$

where TV = tumor volume (cc), ${\mathrm{TV}}_{\mathrm{RX}}$ = tumor volume receiving prescription dose (cc), and = $V_{\mathrm{RX}}$prescription isodose volume (cc). The brain volume receiving 50% of the prescription dose was also analyzed to investigate the model.

For our 40 training cases which were clinically approved, the mean target volume was 6.74 cc (range: 0.02–74.17 cc), the target coverage was 97.8 ± 0.02%, and nCI 1.31 ± 0.15. The mean GM was 4.52 ± 2.00 mm, and the mean brain V_50%_ was 23.17 ± 43.25 cc.

To evaluate the predication accuracy of the model, the 40 plans were evaluated as one group first. Then the fourfold cross‐validation was applied as following: the 40 plans were randomly assigned to four groups, each with ten plans. In turns, three of the groups were used to build the model and the validation was performed using the rest one group. The accuracy of our model prediction was quantified by the mean and the standard deviation of the difference between actual clinical and model predicted values, that is, ΔGM = GM_pred_ ‐ GM_clin_ and ΔV_50%_=V_50%pred_ ‐ V_50%clin_. For the latter, due to the large spread of the absolute PTV volumes, the fractional brain difference of V_50%_ was used instead and therefore fractional brain ${\Delta V}_{50\%}=\frac{V_{50\%pred}‐V_{50\%clin}}{V_{50\%clin}}$.

### 2.C Clinical application of the model

As our model predicts various dose volumes for a given PTV volume, and the dose volumes can be translated to some distances from the target volume boundaries. Therefore, our model allows KBP that automate the optimization process by providing dose constraint at each shell for Cyberknife plan optimization.

From January 2019 to June 2019, 22 intracranial SRS/SRT plans (11 patients, Table 2) were directly generated with the incorporation of our KBP model. The maximum dose constraints of shells during optimization were generated using the model’s prediction based on the PTV volume. Additional OAR constraints were used when OAR was close to the target. The quality of the KBP plans utilizing the model was evaluated and compared with the 40 training plans with manual optimization. The dosimetric indices nCI, GM, and fractional brain ΔV_50%_ were compared here for target coverage and normal tissue sparing analysis. The unpaired t test with Welch’s correction was also used.

**TABLE 2:** KBP test plans’ characteristics.

Prescription	Number of plans *n* ( %) (*N* = 22)	PTV volumes Mean (cc)	OAR involvement Number of Plans
20Gy x 1	10 (45.5%)	0.58 (range: 0.05‐2.22)	0
18Gy x 1	2 (9.0%)	1.44 (range: 0.08‐2.39)	0
8Gy x 3	1 (4.5%)	4.24	0
6Gy x 5	9 (40.9%)	14.50(range: 3.61‐46.91)	2

## 3 Results

### 3.A The univariate regression results

**FIG. 1:**
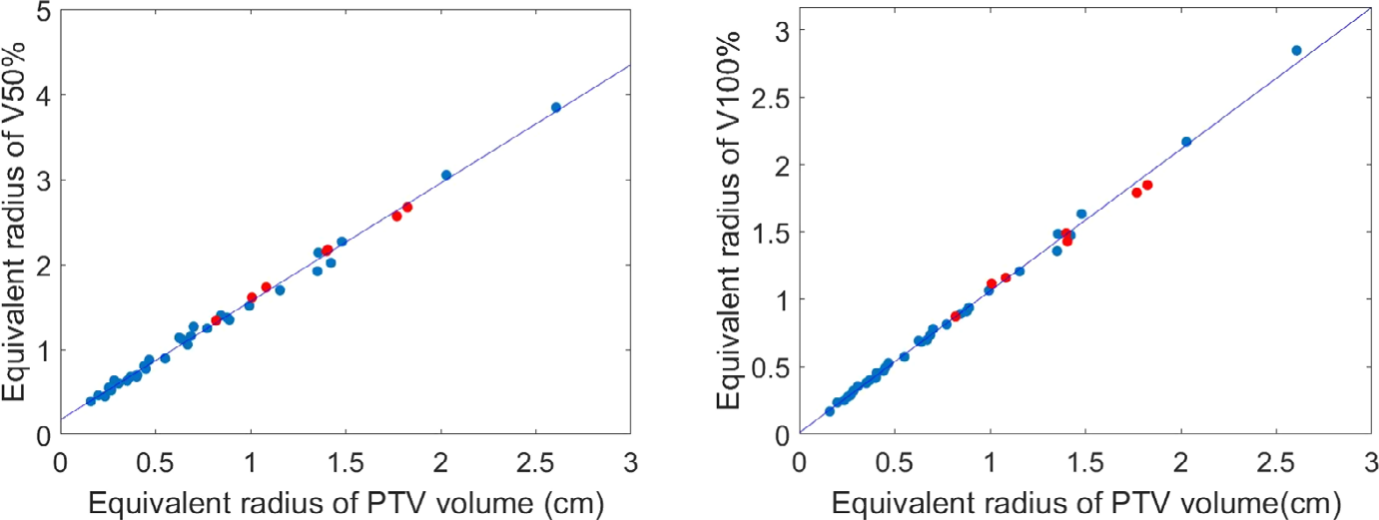
The linear fitting between the equivalent radius of V_50%_ (left), V_100%_ (right) and the equivalent radius of PTV for all 40 patients, R^2^ = 0.995 (left), 0.997 (right), red color indicates plans with OAR involvement.

For the 40 training plans, the R^2^ for the linear fit between r_eq_PTV_ and r_eq_vi_ was 0.985 ± 0.019 for isodose volumes ranging from V_10%_ to V_120%_ (Table 3); particularly, for V_50%_ R^2^ = 0.995 and for V_100%_ R^2^ = 0.997 (Fig. 1).

**TABLE 3:** Linear model fitting parameters for equation (1).

	β	α	R^2^
V_10%_	0.854	2.994	0.951
V_20%_	0.321	2.200	0.984
V_30%_	0.265	1.727	0.993
V_40%_	0.215	1.516	0.994
V_50%_	0.176	1.391	0.995
V_60%_	0.141	1.305	0.995
V_70%_	0.108	1.237	0.995
V_80%_	0.076	1.177	0.996
V_90%_	0.044	1.119	0.996
V_100%_	0.011	1.052	0.997
V_110%_	‐0.025	0.960	0.991
V_120%_	‐0.047	0.795	0.935

### 3.B Prediction accuracy

**FIG. 2:**
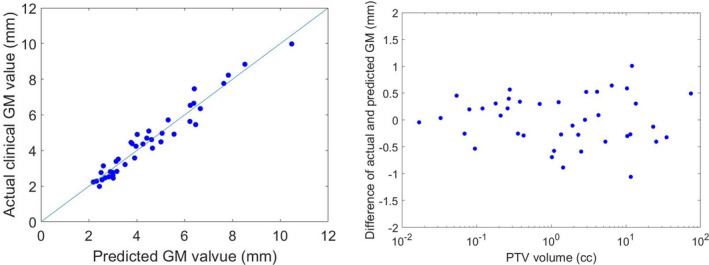
a) The predicted vs. the actual clinical gradient measure (GM) values b) GM prediction error vs. the PTV volume, the mean and the standard deviation for the absolute GM prediction error: 0.38mm and 0.25mm. Due to the large spread of volumes, the x axis was plotted on a log scale for the better illustration.

**FIG. 3:**
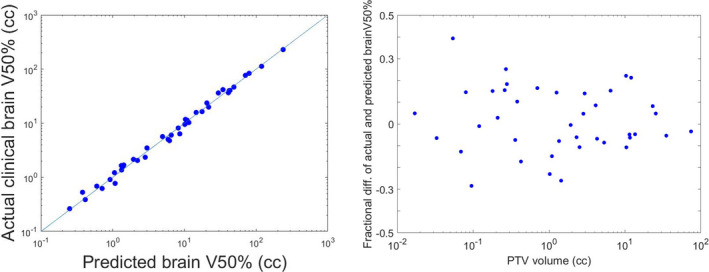
a) the predicted vs. the actual clinical brain V_50%_, b) the fractional brain V_50%_ prediction error vs. the PTV volume, the mean and the standard deviation for the absolute fractional brain V_50%_ error: 0.12 and 0.08. Due to the large spread of volumes, the x axis was plotted on a log scale for the better illustration.

For the 40 training plans, the mean absolute error of predicted vs actual clinical GM was 0.38 ± 0.25 mm (Fig. 2). For V_50%_ predication, the mean absolute error of fractional brain V_50%_ was 0.12 ± 0.08 (Fig. 3). No volume dependence was observed in both GM and fractional brain V_50%_ prediction.

As to the prediction accuracy analysis using the fourfold cross‐validation method, the results were very similar to the above: the average absolute prediction error for ΔGM was 0.39 ± 0.23 mm, and for fractional brain V_50%_ was 0.12 ± 0.08.

### 3.C Clinical application of KBP model

**FIG. 4:**
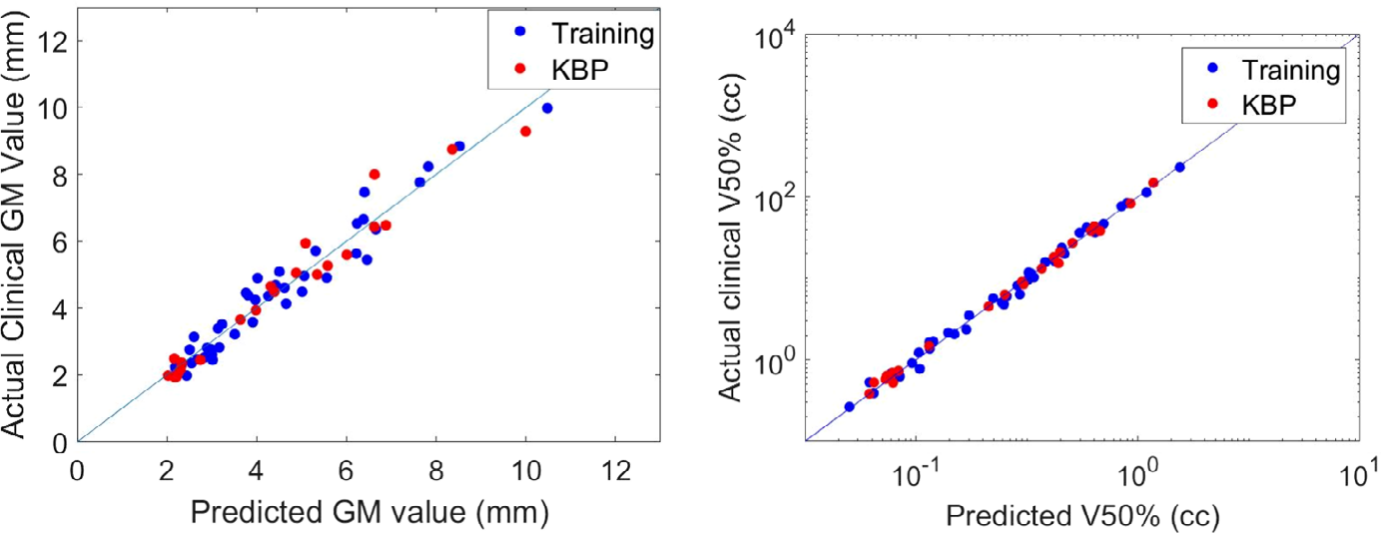
Predicted vs clinical values of GM (mm) and brain V50% (cc) for the training plans and KBP plans.

For the 22 KBP test plans, the mean PTV volume was 6.50 ± 11.0 cc (range: 0.05–46.91 cc), while that was 6.74 cc ± 13.5 cc for the 40 training plans (*P* = 0.94). The PTV volume distribution indicated no significant patient‐specific characteristics between training and testing cases. In 2 of the 22 plans (9.1%), PTV was less than 1 cm away from the brainstem. The prediction error of our model was 0.33 ± 0.31 for GM, and was 0.08 ± 0.07 (Fig. 4) for fractional brain V_50%_.

**FIG. 5:**
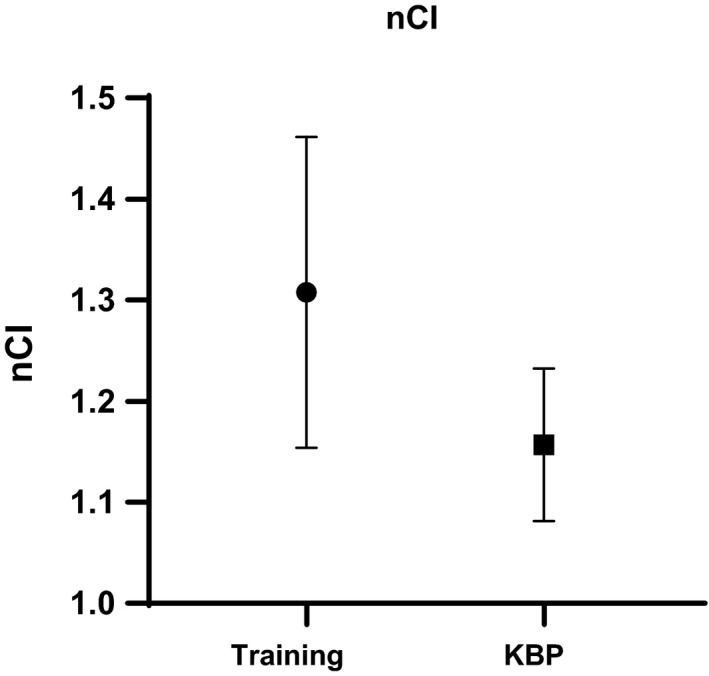
Comparison of nCI between the training plans (n = 40) and KBP test plans (n = 22).

It was found that nCI in the 22 KBP test plans was improved to that of the training plans that were planned with no KBP model: 1.15 ± 0.08 vs. 1.31 ± 0.15 (p < 0.0001, Fig. 5). No difference was found for GM: 4.5 ± 2.3 mm vs 4.5 ± 2.0mm (*P* = 0.97). Slight improvement was observed for the ratio of brain V_50%_/PTV: 4.4 ± 1.2 vs 5.4 ± 2.7 (*P* = 0.0475).

### 3.D Planning efficiency using the model

For 20/22 patients plans, there were no nearby OAR involvement and three to four automated shell constraints (maximum dose) generated from the model were found sufficient in the sequential optimization. All the accepted plans were achieved with one iteration. For the other two plans where OARs were involved, additional constraint for the OAR maximum dose was added, and one or two more iterations was involved depending on the planning goal for the OARs sparing.

## 4 Discussion

We have demonstrated a linear and highly accurate KBP model that predicts CyberKnife SRS/SRT planning dosimetric indices for plan evaluation and optimization. Our work considers both single‐fraction and multi‐fraction plans. The high accuracy of the model was supported by the optimal R^2^ and the small prediction error from the fourfold cross‐validation. The model predicted GM to be within 0.4 mm, and V_50%_ within 12%. The 40 training plans used for modeling consisted a wide range of PTV volumes (range: 0.02 cc‐74.17 cc), and the prediction accuracy was found independent of volume sizes [Figs. 2(b) and 3(b)]. No stratification is needed during our model generation and therefore our model is less sophisticated than previously published studies such as Shiraishi et al’s model.^30^ However, our KBP model for Cyberknife agree with other published KBP models for other modalities in terms of achieving consistent and improved plan quality with higher efficiency.

Our method mainly focuses on PTV coverage and conformity, since not many OARs are usually involved in intracranial SRS/SRT. Although no OAR data were used for the model generation, our modeled linear regression is not only limited to the patients with simple geometry where no OAR is a concern. As highlighted in Fig. 1, the patients with the OAR(s) in close proximity to the PTV also follow the high linear correlation between the equivalent radius of PTV volume and the equivalent radius of V50%/V100%. Therefore, our model can be applied to the intracranial SRS/SRT patients with different geometrical complexity.

We believe that our method helps improve planning efficiency and plan quality in different scenarios. For the cases with close OAR‐PTV proximity, using our model parameters for the shell constraints saves planner’s time by quickly achieving acceptable PTV coverage. Consequently, it becomes more straightforward for the planner to move on to the fine‐tuning the plan in regards to the OAR constraints, which is likely to yield a more desirable OAR sparing. For the cases with no OARs for optimization, our model helps achieve consistent plan quality. According to our experience, when the target structures are away from any OARs, choosing what metrics for plan evaluation may be arbitrary, and very few metrics may end up being used. Due to the different plan evaluation metrics chosen by the physician or the planner, the plan quality may varies significantly even in the same clinic. Our method can efficiently alleviate the problem of inconsistent plan quality, particularly for the uncomplicated cases where no OAR is a concern.

One of our future works is to consider different planning strategies beyond our clinical practice. Due to the limited number of structures in CyberKnife MultiPlan, each target was planned individually for multiple‐target cases in this work. A summation plan was then created to evaluate the total dose to the targets and OARs. There are other planning strategies in CyberKnife MultiPlan for multiple targets, for example: to combine the targets with the same prescription level and then create constraint shells for the combined volume. Also in the manual plans, one to two fixed cone collimators are used for planning to ensure the treatment efficiency. However, with the availability of IRIS collimator and MLCs, the plan dosimetric indices could be different from the ones generated with fixed cone. We are currently working on the KBP model extension to include such planning strategies. Another potential future work is about the optimization approach in newer Cyberknife treatment planning system (i.e., Precision), which is different from the discussed sequential optimization. It will be interesting to investigate the difference of the KBP models of the two systems in the future.

There may be a great potential of our KBP model to be directly adopted in other institutions. Here, we provide our model parameters (Table 3) for the reference purpose to the readers who are interested in using this model. On the other hand, the readers are encouraged to fit their own model based on their own training plans. In addition, our methodology is also applicable to other modalities such as GammaKnife or Linac‐based stereotactic treatments and to other body sites. Our future work also includes the investigation of the model parameters variations between institutions, modalities, and body sites.

## 5 Conclusion

Our work demonstrated a linear and highly accurate knowledge‐based model that predicts CyberKnife SRS/SRT planning dosimetric indices. The developed model helps automate planning process by generating shell constraints based on PTV volumes, which yield comparable or improved quality plans and enhanced efficiency, comparing to the conventional manual planning.

## Conflict of interest

None.

## Author contribution statement

**Suhong Yu** was involved in conceptualization, methodology, software, data curation, and draft preparation. Huijun Xu was involved in writing and revising. Yin Zhang was involved in draft preparation. Xin Zhang was involved in data collection. Michael Dyer, Minh Tam Truong, and, Ariel Hirsh were involved in reviewing and editing. Heming Zhen was involved in software, methodology, and reviewing and editing.
